# Evaluating the Immunogenicity of Protein Drugs by Applying *In Vitro* MHC Binding Data and the Immune Epitope Database and Analysis Resource

**DOI:** 10.1155/2013/467852

**Published:** 2013-10-08

**Authors:** Sinu Paul, Ravi V. Kolla, John Sidney, Daniela Weiskopf, Ward Fleri, Yohan Kim, Bjoern Peters, Alessandro Sette

**Affiliations:** La Jolla Institute for Allergy and Immunology, 9420 Athena Circle, La Jolla, San Diego, CA 92037, USA

## Abstract

The immune system has evolved to become highly specialized in recognizing and responding to pathogens and foreign molecules. Specifically, the function of HLA class II is to ensure that a sufficient sample of peptides derived from foreign molecules is presented to T cells. This leads to an important concern in human drug development as the possible immunogenicity of biopharmaceuticals, especially those intended for chronic administration, can lead to reduced efficacy and an undesired safety profile for biological therapeutics. As part of this review, we will highlight the molecular basis of antigen presentation as a key step in the induction of T cell responses, emphasizing the events associated with peptide binding to polymorphic and polygenic HLA class II molecules. We will further review methodologies that predict HLA class II binding peptides and candidate epitopes. We will focus on tools provided by the Immune Epitope Database and Analysis Resource, discussing the basic features of different prediction methods, the objective evaluation of prediction quality, and general guidelines for practical use of these tools. Finally the use, advantages, and limitations of the methodology will be demonstrated in a review of two previous studies investigating the immunogenicity of erythropoietin and timothy grass pollen.

## 1. Introduction 

Immunogenicity of drug candidates is a significant concern that requires exhaustive evaluation during drug development to ensure maximum efficacy and optimal safety of administered therapeutics [1–4]. Accordingly, to control or abrogate undesired immune responses it is necessary to have a detailed understanding of drug-specific T cell responses. For example, knowledge of the immunogenicity of specific compounds can identify avenues for inhibiting T cells targeting the drug, thereby impairing B cell activation and the development of drug-specific antibody responses. 

**Figure 1:**
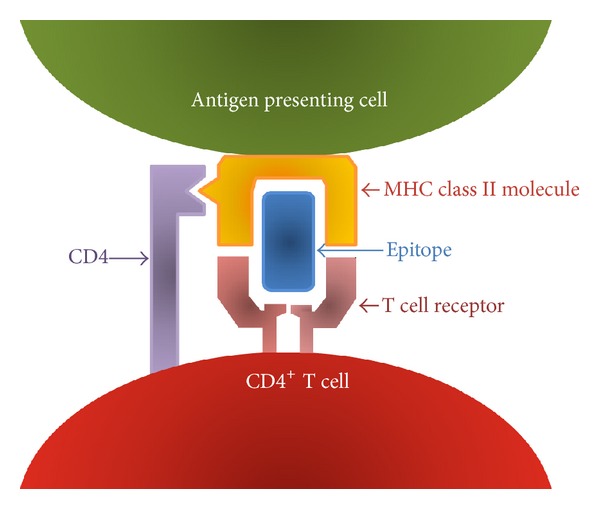
T cells recognize a complex of a peptide fragment and MHC (HLA in humans).

The T cell receptor recognizes a complex formed by a peptide fragment and an MHC molecule (also called Human Leukocyte Antigen, or HLA molecules, in humans) (Figure 1) [5]. This recognition is a necessary event for T cell activation and development of T cell responses. The peptide fragment bound by an HLA molecule, typically generated by proteolytic processing of an antigenic protein, binds in a peptide binding groove within the HLA molecule by engaging the specific side chains of the peptide amino acids. A peptide bound within an HLA molecule and is recognized by a T cell receptor is referred to as an epitope. 

There are two main types of HLA molecules, class I and class II (reviewed in [6]). HLA class I molecules are generally involved in the recognition of proteins synthesized within cells and represent a crucial component in the recognition of viruses and intracellular bacteria. By contrast, HLA class II molecules are involved in the presentation of exogenously derived proteins, including biologic therapeutics, and therefore will be the primary focus of the discussions below. 

HLA class II molecules are alpha/beta heterodimers encoded by three separate loci: HLA-DR, DP, and DQ. Importantly, the HLA genes encoding for class II (and class I) MHC molecules represent some of the most polymorphic loci in mammals. Indeed, several thousand different allelic variants have been described to date (http://www.imgt.org/). It was recognized early on that the allelic variations cluster in very discrete (hypervariable) regions [7]. When the three-dimensional structure of MHC molecules was described [8], it was demonstrated that these hypervariable regions correspond to specific pockets within the molecule that engage peptide side chains, and that each pocket was associated with a relatively narrow chemical specificity. This feature results in the different allelic variants having somewhat unique binding repertoires. 

The definition of a set of HLA molecules that is most representative of the most common allelic variants in the general population is an important issue to be considered in any study addressing HLA class II restricted immunogenicity. This issue was addressed by a series of previous studies from our laboratory [9, 10] that identified a panel of 25 to 40 different HLA molecules that provide global coverage.

In general, a given HLA class II molecule will bind only about 10% of all possible peptide sequences with high affinity (IC_50_ ≤ 100 nM) [9]. As HLA binding is a prerequisite for T cell immunogenicity, it was recognized almost a quarter century ago that tools that would allow efficient prediction of immunogenic peptides (epitopes) would be of enormous value in understanding and modulating the immune response [11–14]. At present, computational tools for HLA binding predictions are readily available online [15].

As discussed briefly above, when protein and antibody therapeutics are processed as protein antigens, an inappropriate immune response against the respective therapeutics may be induced, thereby reducing efficacy and/or causing safety or toxicology concerns for an affected patient. Examples for such adverse immune responses include the development of antibodies against factor VIII in hemophiliacs [16, 17], calcitonin in patients treated for osteoporosis [18, 19], erythropoietin in patients undergoing therapy for chronic renal failure [20, 21], and IFN-*β* in individuals undergoing treatment for multiple sclerosis [22].

In summary, the polygenic and polymorphic nature of HLA binding and T cell recognition leads to a broad and low threshold of selectivity for activation of the immune response. These features reflect the biological function of HLA molecules in host immunity that ensures that for each pathogen protein, at least some peptides are bound and presented to T cells. The immune system has evolved to minimize the likelihood that any given pathogen might mutate and escape detection at both an individual and population level. These biological facts lay the foundations for the challenge of reducing and/or abolishing the immunogenicity of protein drugs which are, unbeknownst to the immune system, friends and not foe. 

In this review, we will discuss different HLA class II epitope prediction methodologies provided by the Immune Epitope Database and Analysis Resource (IEDB—http://tools.iedb.org/main/) and how they can be utilized to modify the immunogenicity of protein therapeutics to mitigate possible safety risks while maximizing efficacy.

## 2. Different Methodologies Used to Predict HLA Class II Binding

An important feature of MHC class II molecules—distinct from class I molecules—is that the ends of their binding groove are open [23]. As a result, MHC class II molecules can bind peptides of somewhat variable length, typically of 13–25 amino acids [24–26]. At the same time, the bulk of the energy of interaction with the MHC molecule is provided by a peptide core of only about 9 amino acids in length [27, 28]. Thus, unlike MHC class I molecules, an epitope bound to an MHC class II molecule can have multiple potential binding registers within it. Algorithms designed to identify MHC class II epitopes must have the capacity to estimate the predictive score for all potential binding registers and select the best one.

Several different methods have been developed to predict peptides binding to HLA molecules. These approaches include simple binding motifs that identify a few key positions and residues associated with high binding capacity, or more sophisticated computational approaches that develop quantitative matrices and/or utilize hidden Markov models and artificial neural networks. The definition of particular “binding motifs” for MHC molecules (similar peptide sequence and structural features in binders compared to nonbinders) led to the development of the first algorithms for MHC binding predictions [12, 29]. These methods relied on identifying whether a particular peptide matched the specific binding motif for that MHC molecule. However, significant variability in the length of the peptides binding to MHC class II molecules makes alignment of the peptides to the binding groove challenging. 

**Figure 2:**
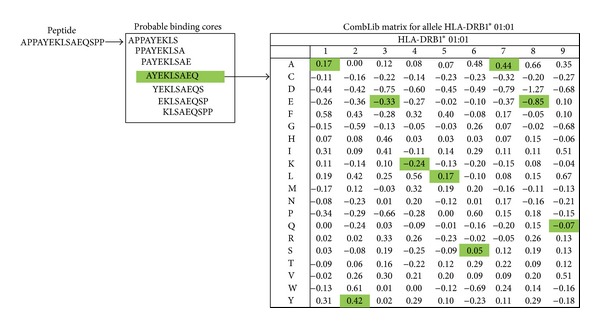
An example of matrices used to generate class II HLA prediction methods. Common prediction methods rely on the derivation of specific matrices that quantify the positive or negative contribution of the 20 different amino acid types to the overall binding affinity for each position.

The availability of large peptide:MHC binding data sets has enabled the use of statistical approaches and the development of matrix-based methods. The position specific scoring matrices (PSSM) quantify, for each position of the peptide, the positive or negative contribution of the 20 different amino acid types to the overall binding affinity in the form of specific matrix scores. The individual scores at each position are then totaled to yield the overall binding score for each peptide, as illustrated in Figure 2. Examples for such matrix-based methods include stabilization scoring matrix align (SMM-align) [11] and average relative binding method (ARB) [30]. Such position-specific quantitative matrices, derived from experimental measurements, have proven over the years to perform reasonably well overall. However, recent applications of more computationally sophisticated machine-learning methods, such as artificial neural networks (ANN), have resulted in appreciable improvements in performance [31, 32]. 

SMM-align, a stabilization matrix alignment method, incorporates information on residues flanking the anchoring amino acids and has improved prediction accuracy [11, 33, 34]. NN-align, an ANN based method, was developed as an extension of SMM-align and also incorporated information on the peptide flanking residues (amino acid composition and length). It also had a new scheme for neural network training that allows for correction of bias in the training data (due to redundant binding core representation) [35]. 

An important development in the field of HLA class II predictions has been the development of a pan-specific method (NetMHCIIpan) that allows for predicting binding even in the case of HLA class II alleles, for which little or no binding data are available. The NetMHCIIpan method is an ANN based method that is trained on quantitative peptide HLA-DR binding data and factors specific information pertaining to the peptide-binding core, peptide flanking residues, and the HLA-DR residues estimated to be within interaction distance of the bound peptide [36]. It is capable of providing quantitative predictions of peptides binding to all HLA-DR molecules with known protein sequence. An updated and faster version of the pan-specific method will be made publicly available in summer 2013.

### 2.1. IEDB as a Resource for MHC Class II Binding Prediction

The most comprehensive collection of epitope prediction and analysis tools is hosted by the Immune Epitope Database and Analysis Resource (IEDB—http://www.iedb.org/) [15, 37]. The IEDB is a free online resource hosting experimentally derived epitope information related to allergens, viruses, microbes, autoimmune diseases, and transplantation epitopes. Overall, it hosts over 15,000 curated articles and direct submissions, 100,000 unique epitopes, and specific data derived from over 600,000 different assays, encompassing class I and class II MHC binding, T cell recognition, and antibody responses. Hosts include humans, nonhuman primates, rodents, and other vertebrates. The IEDB analysis resource additionally contains a variety of different predictive tools, including those for prediction of HLA class II binding, and represents the “best in class” of different prediction methodologies that have been developed over the years and described in peer-reviewed literature. 

The MHC class II binding prediction tool in the IEDB makes available a variety of methods, including NN-align and NetMHCIIpan (both neural network based) [35, 36], SMM-align (based on stabilization matrix alignment) [11], matrices based on positional scanning combinatorial libraries [38], Sturniolo et al. [39] and ARB methods [30] (both based on scoring matrix methods), and finally a consensus method [33, 38] based on a combination of NN-align, SMM-align, and CombLib methods (Table 1). 

The availability of so many different methods raises the issue of how to perform a rigorous, quantitative, and transparent evaluation of their accuracy and sensitivity. To address this issue, Wang et al. [38] evaluated quantitative binding data from the Sette and Buus laboratories relating to 26 different class II molecules, with each represented by an average of approximately 1,500 different measurements. Based on AROC values (area under the receiver operating characteristic curves), a metric used to measure the performance of MHC class II binding prediction tools [38, 40], the top individual scoring methods were in general ARB, SMM-align, and NetMHC, with AROC values in the 0.76 to 0.85 range [38]. A consensus method, based on the median rank of the top 3 methods available for each allele, was determined to be most effective with AROC values of 0.89. Accordingly, while any individual method may be selected, the consensus method has been selected by the IEDB as the default. The consensus method covers 53 DR alleles, and the development of NetMHCIIpan extends coverage to several hundred. Additional recent developments have greatly expanded coverage to include the DQ and DP loci.

To predict class II HLA binding using the default IEDB method, (the consensus method) the user must provide the protein sequence, choose the desired alleles, and select a prediction method. If the specified allele is not available under the consensus method, the NetMHCIIpan method is chosen by default. Upon submission, the tool breaks the protein sequence into all possible 15 mer peptides and then predicts the binding affinity (typically in terms of IC_50_ nM, where lower predicted IC_50_ values are considered better binders) for each peptide. Finally, a percentile rank is generated by comparing the peptide's predicted binding affinity against that of a large set of 15 mers randomly selected from the SWISS-PROT database. Percentile scores provide a uniform scale allowing comparisons across different predictors. A lower percentile rank indicates higher affinity. In the case of the consensus method, the median percentile rank of the three methods is used to generate the consensus percentile rank. 

Selection of predicted binders can be done based on the percentile rank or MHC binding affinity. The IEDB currently recommends making selections based on a consensus percentile rank of the top 10%. Alternatively, selecting peptides predicted to bind at 1,000 nM is also supported by experimental data [41]. In addition to using IEDB generated percentile rank and MHC binding affinity, other alternate approaches for selecting binders can also be utilized depending on a user's needs. For example, if there are too few or too many predicted binders based on the recommended threshold and the user needs more numerous or fewer peptides to study, it is advisable to vary the cut-off values and select the desired number of top scoring peptides. Another scenario may be to set a desired percentage within the user's peptide set (irrespective of IEDB percentile rank), if it is desired to study a fixed number of the best predicted peptides.

As previously mentioned, the tool breaks the sequence into all possible 15 mers, generating a set of peptides overlapping by 14 amino acid residues. This leads to several peptides sharing the same 9-residue core being predicted with the same top scores. To solve this problem, two different approaches are utilized. In the first scenario, the user can “preprocess” the sequence to generate 15 mers overlapping by 10 amino acid residues and then perform the prediction on this peptide set. Alternatively, following the predictions, the user can do a “postprocessing” step to remove largely overlapping peptides.

A stand-alone version of the predictive tool is also provided by the IEDB which can be downloaded from the IEDB website. This version has several advantages, including allowing the user to implement tools on his/her own machine. This in turn may allow better handling of large volumes of data (genome scale) and repetitive analyses. This approach can make predictions faster. The stand-alone version is freely available for nonprofit and academic institutions and at a nominal license fee to industry. 

### 2.2. Test Case Scenario 1: Immunogenicity of EPO

Tangri et al. [14] described an analysis of the dominant HLA class II restricted epitopes contained within the EPO sequence whose immunogenicity was linked to rather severe reactions [20, 21]. In this section we will briefly review the main findings of the Tangri study in the context of the discussions above. 

Tangri et al. [14] synthesized a set of overlapping peptides spanning the entire EPO sequence and tested them for binding to common HLA molecules. The results illustrated, as expected, that nearly all peptides will binds at least some of the common HLA molecules and each of the common HLA molecules bind multiple EPO peptides. Thus, generating an EPO protein homolog that is not at all immunogenic is likely an unrealistic goal. Importantly, however, the same results suggest that overall HLA binding patterns can be used to rank protein variants to reduce potential immunogenicity.

In fact, a subsequent series of experiments tested the immunogenicity *in vitro *of the same set of peptides for human lymphocytes and identified two broad, immunogenic regions. Further analysis demonstrated that corresponding peptides were promiscuous; that is, the peptides had the capacity to bind multiple HLA molecules. 

At the same time, HLA class II binding promiscuity was utilized to generate EPO variants associated with lower immunogenicity *in vitro*. Specifically, variant peptides were generated containing substitutions predicted to promote lower promiscuity. It was shown that peptides binding fewer HLA alleles were less immunogenic and that EPO proteins carrying those substitutions were also less immunogenic *in vitro.* Thus, this data demonstrated that promiscuity can be used to rank the relative immunogenicity of protein variants.

Extending these observations, in the case of EPO, if a user desired to use IEDB tools to predict immunogenicity, a set of 31 peptides (15 mers overlapping by 10 residues) would have been generated and entered in the prediction tool. After selection of a set of HLA molecules representative of the general population, a total of 31 peptides × 24 alleles results would be used to select the predicted binding peptides for each allele according to the general guidelines described above. Finally, the number of alleles predicted to bind each epitope would be determined and plotted along the EPO sequence to highlight predicted promiscuous regions. This same strategy could also be utilized to compare the relative predicted binding potential of different proteins and related variants. Taken together, these observations demonstrate how HLA class II predictions may be useful to compare the potential immunogenicity of protein variants and guide the development of biological therapeutics with potentially reduced immunogenicity.

### 2.3. Test Case Scenario 2: Pollen Immunogenicity and Promiscuous Epitopes

An independent series of investigations mapped the dominant epitopes recognized in response to timothy grass pollen allergens. In these studies [42], ten different Phl p proteins were considered (Phl p 1, 2, 3, 4, 5, 6, 7, 11, 12, and 13) and were chosen based on being the targets of IgE antibody responses.

A total of 687 overlapping peptides spanning these allergens were synthesized. Next, peptide pools were tested for reactivity with T cell lines obtained by *in vitro* timothy grass extract restimulation of PBMC from a cohort of allergic patients. As expected, a large fraction of the peptides tested were immunogenic and a total of 43 different antigenic regions were identified. Importantly, it was noted that the 9 most dominant regions accounted for approximately half of the total T cell reactivity. Further analysis demonstrated that bioinformatics prediction of the most promiscuous epitopes also allowed identification of roughly 50% of the total T cell response. 

This data provided the foundation for an approach to identify dominant responses based on prediction of HLA promiscuous binding. This approach has since been utilized to identify epitopes in many other allergen targets and the performance of a genome-wide mapping of tuberculosis-associated epitopes [43–46].

## 3. Conclusions

The biological function of HLA class II molecules is to bind and present exogenous peptides for T cell scrutiny. As such, the polygenic and polyallelic nature of HLA molecules ensures, in general, that each molecule can bind multiple peptides derived from each protein and that each peptide will be bound by some of the many locus and allelic HLA variants. The resulting heterogeneity provides a significant challenge for a pathogen or a biological therapeutic to escape recognition by the immune system. 

Despite its complexity, this issue can be experimentally addressed using the notion that HLA binding capacity can be effectively measured and predicted. A number of HLA class II predictors are freely available online. The breadth of experimentally based algorithms includes mostly human molecules; however, some murine predictors are also available. While the accuracy of class II predictors is still lower than that of class I predictors (current average AROC = 0.87 ± .005), substantial improvements have been made over recent years (in 2006, average AROC = 0.76 ± .005); these advances include the development of PAN predictors, enhanced speed, and updated predictive tools. Additionally, a new version is planned for release on the IEDB later in 2013.

While the goal of generating biologic-based therapeutics that pass undetected by HLA class II molecules is challenging, prediction algorithms can be utilized to identify promiscuous HLA class II binders that have been demonstrated to represent dominant T cell epitopes. In addition, HLA class II predictions are useful to compare the potential immunogenicity of protein variants and guide the development of efficacious therapeutics with potentially reduced immunogenicity.

## Figures and Tables

**Table 1:** The MHC class II prediction tools available at IEDB. A user may choose from one of the seven prediction methods provided. The consensus method is used as the default method and is composed of three of the most successful individual prediction methods.

Methods	Prediction based on	Reference
Consensus	Combination of NN-align, SMM-align and CombLib	Wang et al., 2010 [38]
NetMHCIIpan	Artificial neural network	Nielsen et al., 2010 [31]
NN-align	Artificial neural network	Nielsen and Lund, 2009 [35]
SMM-align	Stabilization matrix alignment	Nielsen et al., 2007 [11]
Combinatorial library	Position scanning combinatorial libraries	Wang et al., 2008 [33] Wang et al., 2010 [38]
Sturniolo	Scoring matrix based	Sturniolo et al., 1999 [39]
ARB	Average relative binding	Bui et al., 2005 [30]

## References

[1] Kropshofer H, Singer T (2006). Overview of cell-based tools for pre-clinical assessment of immunogenicity of biotherapeutics. *Journal of Immunotoxicology*.

[2] Wolbink GJ, Aarden LA, Dijkmans BAC (2009). Dealing with immunogenicity of biologicals: assessment and clinical relevance. *Current Opinion in Rheumatology*.

[3] Brinks V, Jiskoot W, Schellekens H (2011). Immunogenicity of therapeutic proteins: the use of animal models. *Pharmaceutical Research*.

[5] Murphy K (2011). *Janeway’s Immunobiology*.

[6] Paul WE (2013). *Fundamental Immunology*.

[7] Benoist CO, Mathis DJ, Kanter MR (1983). Regions of allelic hypervariability in the murine A_*α*_ immune response gene. *Cell*.

[8] Brown JH, Jardetzky TS, Gorga JC (1993). Three-dimensional structure of the human class II histocompatibility antigen HLA-DR1. *Nature*.

[9] Greenbaum J, Sidney J, Chung J, Brander C, Peters B, Sette A (2011). Functional classification of class II human leukocyte antigen (HLA) molecules reveals seven different supertypes and a surprising degree of repertoire sharing across supertypes. *Immunogenetics*.

[10] McKinney DM, Southwood S, Hinz D (2013). A strategy to determine HLA class II restriction broadly covering the DR, DP, and DQ allelic variants most commonly expressed in the general population. *Immunogenetics*.

[11] Nielsen M, Lundegaard C, Lund O (2007). Prediction of MHC class II binding affinity using SMM-align, a novel stabilization matrix alignment method. *BMC Bioinformatics*.

[12] Sette A, Buus S, Appella E (1989). Prediction of major histocompatibility complex binding regions of protein antigens by sequence pattern analysis. *Proceedings of the National Academy of Sciences of the United States of America*.

[13] Brusic V, Rudy G, Honeyman G, Hammer J, Harrison L (1998). Prediction of MHC class II-binding peptides using an evolutionary algorithm and artificial neural network. *Bioinformatics*.

[14] Tangri S, Mothé BR, Eisenbraun J (2005). Rationally engineered therapeutic proteins with reduced immunogenicity. *The Journal of Immunology*.

[15] Kim Y, Ponomarenko J, Zhu Z (2008). Immune epitope database analysis resource. *Nucleic Acids Research*.

[16] Bristol JA, Gallo-Penn A, Andrews J, Idamakanti N, Kaleko M, Connelly S (2001). Adenovirus-mediated factor VIII gene expression results in attenuated anti-factor VIII-specific immunity in hemophilia A mice compared with factor VIII protein infusion. *Human Gene Therapy*.

[17] Kulkarni R, Aledort LM, Berntorp E (2001). Therapeutic choices for patients with hemophilia and high-titer inhibitors. *American Journal of Hematology*.

[18] Reginster JY, Gaspar S, Deroisy R, Zegels B, Franchimont P (1993). Prevention of osteoporosis with nasal salmon calcitonin: effect of anti-salmon calcitonin antibody formation. *Osteoporosis International*.

[19] Grauer A, Ziegler R, Raue F (1995). Clinical significance of antibodies against calcitonin. *Experimental and Clinical Endocrinology & Diabetes*.

[20] Casadevall N, Nataf J, Viron B (2002). Pure red-cell aplasia and antierythropoietin antibodies in patients treated with recombinant erythropoietin. *The New England Journal of Medicine*.

[21] Gershon SK, Luksenburg H, Coté TR (2002). Pure red-cell aplasia and recombinant erythropoietin. *The New England Journal of Medicine*.

[22] Deisenhammer F, Mayringer I, Harvey J (2000). A comparative study of the relative bioavailability of different interferon beta preparations. *Neurology*.

[23] Stern LJ, Brown JH, Jardetzky TS (1994). Crystal structure of the human class II MHC protein HLA-DR1 complexed with an influenza virus peptide. *Nature*.

[24] Rudensky AY, Preston-Hurlburt P, Hong S, Barlow A, Janeway CA (1991). Sequence analysis of peptides bound to MHC class II molecules. *Nature*.

[25] Chicz RM, Urban RG, Lane WS (1992). Predominant naturally processed peptides bound to HLA-DR1 are derived from MHC-related molecules and are heterogeneous in size. *Nature*.

[26] Castellino F, Zhong G, Germain RN (1997). Antigen presentation by MHC class II molecules: invariant chain function, protein trafficking, and the molecular basis of diverse determinant capture. *Human Immunology*.

[27] Rammensee H, Friede T, Stevanović S (1995). MHC ligands and peptide motifs: first listing. *Immunogenetics*.

[28] Reche PA, Glutting J-P, Zhang H, Reinherz EL (2004). Enhancement to the RANKPEP resource for the prediction of peptide binding to MHC molecules using profiles. *Immunogenetics*.

[29] Rotzschke O, Falk K, Stevanovic S, Jung G, Walden P, Rammensee HG (1991). Exact prediction of a natural T cell epitope. *European Journal of Immunology*.

[30] Bui HH, Sidney J, Peters B (2005). Automated generation and evaluation of specific MHC binding predictive tools: ARB matrix applications. *Immunogenetics*.

[31] Nielsen M, Lund O, Buus S, Lundegaard C (2010). MHC Class II epitope predictive algorithms. *Immunology*.

[32] Lundegaard C, Hoof I, Lund O, Nielsen M (2010). State of the art and challenges in sequence based T-cell epitope prediction. *Immunome Research*.

[33] Wang P, Sidney J, Dow C, Mothé B, Sette A, Peters B (2008). A systematic assessment of MHC class II peptide binding predictions and evaluation of a consensus approach. *PLoS Computational Biology*.

[34] Lin HH, Zhang GL, Tongchusak S, Reinherz EL, Brusic V (2008). Evaluation of MHC-II peptide binding prediction servers: applications for vaccine research. *BMC Bioinformatics*.

[35] Nielsen M, Lund O (2009). NN-align, an artificial neural network-based alignment algorithm for MHC class II peptide binding prediction. *BMC Bioinformatics*.

[36] Nielsen M, Justesen S, Lund O, Lundegaard C, Buus S (2010). NetMHCIIpan-2.0—improved pan-specific HLA-DR predictions using a novel concurrent alignment and weight optimization training procedure. *Immunome Research*.

[37] Salimi N, Fleri W, Peters B, Sette A (2012). The immune epitope database: a historical retrospective of the first decade. *Immunology*.

[38] Wang P, Sidney J, Kim Y (2010). Peptide binding predictions for HLA DR, DP and DQ molecules. *BMC Bioinformatics*.

[39] Sturniolo T, Bono E, Ding J (1999). Generation of tissue-specific and promiscuous HLA ligand databases using DNA microarrays and virtual HLA class II matrices. *Nature Biotechnology*.

[40] Swets JA (1988). Measuring the accuracy of diagnostic systems. *Science*.

[41] Southwood S, Sidney J, Kondo A (1998). Several common HLA-DR types share largely overlapping peptide binding repertoires. *The Journal of Immunology*.

[42] Oseroff C, Sidney J, Kotturi MF (2010). Molecular determinants of T cell epitope recognition to the common timothy grass allergen. *The Journal of Immunology*.

[43] Oseroff C, Sidney J, Tripple V (2012). Analysis of T cell responses to the major allergens from german cockroach: epitope specificity and relationship to IgE production. *The Journal of Immunology*.

[44] Oseroff C, Sidney J, Vita R (2012). T cell responses to known allergen proteins are differently polarized and account for a variable fraction of total response to allergen extracts. *The Journal of Immunology*.

[45] Schulten V, Greenbaum JA, Hauser M (2013). Previously undescribed grass pollen antigens are the major inducers of T helper 2 cytokine-producing T cells in allergic individuals. *Proceedings of the National Academy of Sciences*.

[46] Arlehamn CSL, Gerasimova A, Mele F (2013). Memory T cells in latent mycobacterium tuberculosis infection are directed against three antigenic islands and largely contained in a CXCR3^+^CCR6^+^ Th1 subset. *PLoS Pathogens*.

